# Local tumor control probability to evaluate an applicator‐guided volumetric‐modulated arc therapy solution as alternative of 3D brachytherapy for the treatment of the vaginal vault in patients affected by gynecological cancer

**DOI:** 10.1120/jacmp.v14i2.4075

**Published:** 2013-03-04

**Authors:** Piernicola Pedicini, Lidia Strigari, Rocchina Caivano, Alba Fiorentino, Giorgia Califano, Antonio Nappi, Giuseppina Improta, Giovanni Storto, Mariella Cozzolino, Costanza Chiumento, Vincenzo Fusco, Andrea Vavassori, Marcello Benassi, Roberto Orecchia, Marco Salvatore

**Affiliations:** ^1^ I.R.C.C.S. Regional Cancer Hospital C.R.O.B Rionero‐in‐Vulture; ^2^ Regina Elena National Cancer Institute Roma; ^3^ I.E.O. European Institute of Oncology Milan; ^4^ Scientific Institute of Tumours of Romagna I.R.S.T. Meldola; ^5^ University of Milan Milan; ^6^ I.R.C.C.S. SDN Foundation Naples Italy

**Keywords:** VMAT, 3D brachytherapy, EUD, TCP

## Abstract

The purpose of this study was to evaluate the applicator‐guided volumetric‐modulated arc therapy (AGVMAT) solution as an alternative to high‐dose‐rate brachytherapy (HDR‐BRT) treatment of the vaginal vault in patients with gynecological cancer (GC). AGVMAT plans for 51 women were developed. The volumetric scans used for plans were obtained with an implanted CT‐compatible vaginal cylinder which provides spatial registration and immobilization of the gynecologic organs. Dosimetric and radiobiological comparisons for planning target volume (PTV) and organs at risk (OARs) were performed by means of a dose‐volume histogram (DVH), equivalent uniform dose (EUD), and local tumor control probability (LTCP). In addition, the integral dose and the overall delivery time, were evaluated. The HDR‐BRT averages of EUD and minimum LTCP were significantly higher than those of AGVMAT. Doses for the OARs were comparable for the bladder and sigmoid, while, although HDR‐BRT was able to better spare the bowel, AGVMAT provided a significant reduction of $d_{2\text{cc}}$, $d_{1\text{cc}}$, and $d_{\max}$ (p<0.01) for the rectum. AGVMAT integral doses were higher than HDR‐BRT with low values in both cases. Delivery times were about two or three times higher for HDR‐BRT with respect to the single arc technique (AGVMAT1) and dual arc technique (AGVMAT2), respectively. The applicator‐guided volumetric‐modulated arc therapy seems to have the potential of improving rectum avoidance. However, brachytherapy improves performance in terms of PTV coverage, as demonstrated by a greater EUD and better LTCP curves.

PACS number: 87

## I. INTRODUCTION

External beam radiotherapy (EBRT) of GC is generally used for patients with FIGO stage I‐IVA to treat the original tumor site and the regional lymph nodes at high risk for microscopic invasion. HDR‐BRT is performed to administer a high dose to unresected or residual primary tumor in the vaginal vault.^(^ ^1^ ^,^ ^2^ ^,^ ^3^ ^)^

The technical advantage of HDR‐BRT is the steep dose falloff from the ${\;}^{192}\text{Ir}$ source that is placed in the vagina applicator by a remote afterloader.

Pötter et al.^(^ ^4^ ^)^ identified the values that have clinical relevance for the tolerance of the OARs, such as rectum, bladder, and sigmoid, while several other papers have been published which investigate intensity‐modulated radiotherapy (IMRT) in combination or as an alternative to HDR‐BRT.^(^ ^5^ ^,^ ^6^ ^,^ ^7^ ^,^ ^8^ ^)^

An original solution was introduced by Low et al.^(^ ^9^ ^)^ and then by Wahab et al.^(^ ^10^ ^)^ for delivering highly conformal dose distributions to cervical cancer (CC) tumors using external beam IMRT. This method, termed applicator‐guided intensity‐modulated radiation therapy (AGIMRT), uses an applicator placed in the vagina to provide spatial registration and immobilization of the gynecologic organs. In general, the main reason for the use of the applicator was to localize the fornices, cervix, and uterus, as well as surrounding organs reproducibly positioned around the applicator. This was an attractive solution for institutions without the brachytherapy technology because AGIMRT is expected to produce similar outcomes to those obtained by brachytherapy.

In the works of Low and Wahab and their colleagues, the brachytherapy was compared with IMRT and AGIMRT from the dosimetric point of view. AGIMRT seemed to have the capability to reduce the doses to the rectum and bladder, while maintaining highly reproducible and accurate internal organ registration found with brachytherapy. However, in those papers, it was not clear whether the dose distributions obtained by AGIMRT on PTV would give the same therapeutic results of HDR‐BRT.

In our previous work,^(^ ^11^ ^)^ we investigated the benefits by using a solution similar to AGIMRT, exploiting the greater capacity for modulation of the dose obtained by volumetric‐modulated arc therapy (VMAT) instead of conventional IMRT with fixed beams (this solution will be referred to as AGVMAT in this paper). The final goals were to produce similar or better dose distributions compared to HDR‐BRT planning, and obtain therapeutic solutions in a delivery time comparable with those of HDR‐BRT.

As a result, the dose reduction to the rectum was confirmed, while a better PTV coverage was performed by HDR‐BRT in terms of EUD.

However, because EUD represents the biological averages of the values generated by cells exposed to different doses in a very small region of tissue (high gradients of doses), this paper aims to introduce an additional tool for analysis to support radiobiological data interpretation, and then determine if there is a real equivalence in terms of the therapeutic efficacy of techniques. In particular, we calculated the local tumor control probability (LTCP) that permits an evaluation of the local reduction in TCP due to the possible spatial variation in tumor cell density in the small region which must be treated by radiation.

Moreover, in this paper, we used a slightly more modified AGVMAT approach than that adopted in our previous study, using the CT images and contours of a larger cohort of patients, and adopting a different dose prescription.

## II. MATERIALS AND METHODS

### A. Patient selection

From October 2008 to May 2011, 51 women aged over 18 (range 40–81, median 62 years) with histologically proven squamous cell carcinoma, adenocarcinoma, or adenosquamous carcinoma of the cervix and endometrium (endometrial cancer (EC)), were retrospectively selected. Of these, ten patients (19.6%) were affected by CC and 41 patients (80.4%) by EC. All patients were staged according to the FIGO definition, received hysterectomy, postoperative EBRT, and subsequently a vaginal vault HDR‐BRT to reduce the risk of vault recurrence. The patient characteristics are shown in Table 1.

**Table 1:** Main descriptive data of the patient selection.

*Histology*	
EC	41 pts (80.4%)
CC	10 pts (19.6%)
*Figo Stage*	
IB	11 pts (21.6%)
IC	15 pts (29.4%)
IIA	5 pts (9.8%)
IIB	9 pts (17.6%)
IIIA	11 pts (21.6%)
*EBRT Dose*	
45 Gy (25fr)	14 pts (27.4%)
50.4 Gy (28fr)	37 pts (72.6%)
*HDR‐BRT dose*	
4 βy 6 Gy	14 pts (27.4%)
3 βy 6 Gy	37 pts (72.6%)

### B. Classical planning technique

A 3D conformal radiotherapy (3D CRT) plus HDR‐BRT was performed for all patients selected.

3D CRT was planned on residual tumor (or surgical bed) plus pelvic lymph nodes with a total dose of 45–50.4 Gy (1.8 Gy/day); only 14 patients received 45 Gy. All 3D CRT plans were developed employing four 15 MV photon beams by a Varian Clinac 2100DX equipped with a 120‐leaf Millennium Multileaf Collimator (MLC) (Varian Medical Systems, Palo Alto, CA).

The HDR‐BRT boost of dose was prescribed for CTV defined as 0.5 cm around the vaginal dome cylinder for a length of 4 cm without margins (PTV=CTV).^(^ ^3^ ^)^ HDR‐BRT was performed one week after 3D CRT, delivering 6 Gy/week. The boost of dose was delivered with 4 or 3 fractions (fr) to the patients receiving the prophylactic dose of 45 Gy and 50.4 Gy, respectively.

Treatment plans were developed by a treatment planning system (TPS) (Oncentra MasterPlan (v3.3); Nucletron BV, Veenendaal, The Netherlands), using a suitable diameter with a vaginal applicator to optimize the dose distribution and to reduce the mucosal dose. All patients were treated by a microSelectron v3 (Nucletron) that used a ${\;}^{192}\text{Ir}$ source with air kerma strength of 43190 $\text{cGy}•{\text{cm}}^{2}/h$ and an apparent source activity of 10.58 Ci at the time of calibration.

### C. AGVMAT plans

Theoretical AGVMAT plans were generated for all patients previously treated by HDR‐BRT at our Institution.

Similar to the HDR‐BRT schedules, 3 or 4 fractions of 6 Gy/fr were planned by AGVMAT with an interval of one week after 3D CRT.

The AGVMAT plans were developed by RapidArc technique (Varian Medical System, Palo Alto, CA) using volumetric scans with an implanted CT‐compatible vaginal cylinder. The CTV was drawn with the same criterion adopted for HDR‐BRT adding a minimal PTV margin. This is due, on the one hand, because the vaginal cylinder reproduced the same shape as the vaginal vault for each application, while it reduced the setup uncertainty by making the IGRT repositioning of the vaginal vault clearly visible in the cone‐beam CT images. Furthermore, the vaginal applicator was locked into the external immobilization device, reducing the potential intrafractional patient motion. Thus, based on the phantom simulation performed in our Institution, the margin between CTV and PTV as a uniform expansion of 2 mm in all directions was assumed. Overall, exploiting such reproducibility and accuracy, this approach was a pure technique of external irradiation with some important advantages from brachytherapy. To obtain the exact level of dose modulation, the instantaneous dose rate (DR), the MLC leaves' position, and the rotational speed of the gantry were continuously varied by the optimizer. Two different plans were developed: AGVMAT1, consisting of a single arc from 180.1° to 179.9° with clockwise rotation; and AGVMAT2, consisting of two coplanar arcs from 180.1° to 179.9° and from 179.9° to 180.1° with clockwise and counterclockwise rotation, respectively. The application of two coplanar arcs is for the purpose of investigating the increase of the modulation factor during optimization.^(^ ^12^ ^)^ To minimize the tongue‐and‐groove effect, the collimator was fixed at 45° in AGVMAT1 and at 45° and 315° for each arc in AGVMAT2, respectively. Plans were optimized selecting a maximum DR of 600 MU/min and a maximum of 2000 MUs.

### D. Evaluation tools

The AGVMAT dose distributions were compared against HDR‐BRT at various levels by means of standard DVHs.

Because of the typical shape of dose distribution with radioactive seeds, characterized by a considerable falloff of dose with distance from the central position, the dose distributions with HDR‐BRT and EBRT were very different. However, this effect was limited to the target shape, which is a ring with a thickness of only 0.5 cm around the vaginal applicator.

HDR‐BRT plans were normalized on the outer surface of the CTV (coinciding with the PTV in this case), while AGVMAT plans were normalized on the outer surfaces of the PTV. With this premise, we evaluated the mean and maximum doses for the PTV from DVH.

Moreover, from the radiobiological point a view, a comparison of the surviving fraction S of cells in the tissue exposed to a total radiation dose D was introduced.^(^ ^13^ ^)^ The cell survival probability is given by:

(1) S=exp(−E)

where the biological effect of radiation effect *E* can be expressed as:

(2) E=n⋅d⋅(α+β⋅d)

Here α and β are the parameters describing the intrinsic cell radiosensitivity, and *d* and *n* represent the dose per fraction and the number of fractions, respectively.

To take into account the dose heterogeneity, we previously calculated the survival fraction based on DVH using the following:

(3) $$S=\sum_{j}\frac{V_{j}}{V_{0}}S(D_{j})$$

where $V_{0}$ is the PTV volume and $V_{j}$ is the subvolume corresponding to dose bin $D_{j}$ in the DVH. Each $S\left(D_{j}\right)$ is obtained from Eq. (1) for different $D_{j}$.

Subsequently, to compare different dose distributions, an EUD evaluation was made.^(^ ^14^ ^)^ The surviving fraction S, resulting from any dose delivery scheme, can be formulated as:

(4) $$S=\text{exp}\left(-EUD\cdot (\alpha +\beta \cdot d)+0.5\gamma \frac{EUD}{d}\right)$$

where γ is the effective tumor cell repopulation rate ($\gamma =\ln2/T_{d},T_{d}$ is the tumor cell doubling time).

In this paper, we adopted a γ equal to 0.1 ($T_{d}∼6.6$ days^(^ ^15^ ^)^.

From Eq. (2), the corresponding EUD that results in the surviving fraction S can be calculated by the following:^(^ ^16^ ^–^ ^17^ ^)^

(5) $$EUD=\frac{-\ln(S)}{\alpha +\beta \cdot d-0.5\gamma /d}$$

In these equations, it was implicitly assumed that the density of tumor clonogens is constant throughout the tumor, thereby indicating with N=ρ×V the initial number of clonogens (ρ=constant density and V=volume). The tumor control probability (TCP) with clonogen proliferation is calculated from the cell surviving fraction S shown in Eq. (3) using the Poisson's hypothesis:

(6) $$TCP=e^{-\rho V\cdot S}$$

Most calculations of the biological effect of radiation on tumors assume the uniform clonogenic cell density even if a nonuniform dose distribution is taken into account. In practice, tumors will almost certainly have a nonuniform clonogenic cell density. Therefore we used the concept of LTCP, introduced by Webb and Nahum,^(^ ^18^ ^)^ to take into account a possible spatial variation in tumor cell density ρ. Then, by means of LTCP curves, we evaluated the local reduction in TCP, depending on the spatial variation in tumor cell density and on the dose distributions from different techniques.

Hence, in order to calculate the LTCP, the following expression for ρ was placed within the Eq. (6):

(7) $$\rho (r)=\rho_{0}\cdot e^{-\frac{r}{r_{0}}}$$

**Figure 1:**
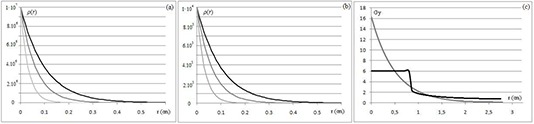
Profiles of tumor cell density ρ(r) (${\text{cells}/\text{cm}}^{3}$) decreasing exponentially in the microscopic extension region with regard to $r_{0}=1\text{mm}$ (black), $r_{0}=0.62\text{mm}$ (gray), $r_{0}=0.31\text{mm}$ (light gray), and $\rho_{0}=10^{7}\text{cells}/{\text{cm}}^{3}$ (a) or $\rho_{0}=10^{4}\text{cells}/{\text{cm}}^{3}$ (b). Schematic dose profiles for HDR‐BRT (gray) and AGVMAT (black) in the CTV region (c).

Here *r* and $\rho_{0}$ represent the distance and the clonogen density at cylinder surface, respectively, while $r_{0}$ defines the steepness of density decrease (Fig. 1(a)). Our hypothesis was that p decreased exponentially in the microscopic extension region with a very wide and clinically likely range of values, from $10^{4}\text{cells}/{\text{cm}}^{3}$ to $10^{7}\text{cells}/{\text{cm}}^{3}$,^(^ ^19^ ^)^ to simulate different values of subclinical clonogens in the microscopic extension region and a residual of clonogens in the tumor bed, respectively. Likewise, various decay coefficients ($r_{0}=1\;\text{mm}$, 0.62 mm, 0.31 mm, and ∞) were adopted to simulate different shapes of density reduction along the distance from the surface of the vaginal applicator. The combinations of these parameters also include the simulation of suspected tumor cells beyond the CTV, which would require an expansion of CTV itself.

LTCP dose responses for all the combinations of tumor cell density and decay coefficients were then studied.

Doses at volumes 2 cc, 1 cc and minimum ($d_{\max}$) were evaluated on DVHs for rectum, bladder, and sigmoid colon. Only $d_{\max}$ was assessed for bowel. In particular, the doses with a 2 cc volume were evaluated for direct comparison with the tolerance of doses given in the work of (GYN) GEC‐ESTRO.^(^ ^4^ ^)^ For patients who received EBRT with 25 fractions of 1.8 Gy (total 45 Gy), tolerances of 4.4 Gy/fr (total 4 fractions) to 2 cc of rectum and sigmoid and 6.2 Gy/ fr to 2 cc of bladder, were adopted. For patients who received 28 fractions of 1.8 Gy/fr (total 50.4 Gy), tolerances of 4.8 Gy/fr to 2 cc of rectum and sigmoid and 7.0 Gy/fr to 2 cc of bladder, were adopted.^(^ ^20^ ^,^ ^21^ ^)^ These values were obtained transforming the tolerances given in the work of Pötter and colleagues, as equivalent dose delivered at 2 Gy/fr (EQD2),^(^ ^22^ ^)^ after subtracting 45 Gy or 50.4 Gy EQD2. Then, using the linear quadratic model, the remaining doses in 4 and 3 fractions (using α/β=3Gy), were obtained. Differences between techniques of doses to bowel were directly compared by using of the DVH.

Moreover, in order to compare the integral dose of radiation resulting from the different plan modalities, a structure was defined to evaluate the mean dose. For the delineation of this structure, we took into account a different extension, in the cranial–caudal direction, of the CT slices. To standardize the ROI in all cases analyzed, the structure coincided with the body until 6 cm from the vaginal vault in the cranial direction and 6 cm in the caudal direction with exclusion of the PTV volume.

Finally, the time needed to deliver the entire treatment by AGVMAT and the mean time needed by HDR‐BRT were compared. For this purpose, we took into account the length of HDR‐BRT treatment that depends on the discharged ${\;}^{192}\text{Ir}$ source from the date of replacement thereof in the microSelectron v3. A source of 10 Ci (370 GBq) replaced four times a year has an activity at the end of the third month, which is the time of its replacement, of 4.3 Ci (159 GBq). Therefore, the same treatment delivered using a ${\;}^{192}\text{Ir}$ source placed in the vaginal vault would last only 4–5 minutes in the early days after the replacement, while it would be 9–11 minutes after three months.

### E. Statistical analysis

The standard Fisher test was made with a Bonferroni correction because of the comparison between groups. The p‐values under 0.01 were assumed as significant, while p‐values between 0.01 and 0.05 were considered as a trend. All information about the statistical analysis can be found in Pearson et al.^(^ ^23^ ^)^

## III. RESULTS

**Figure 2:**
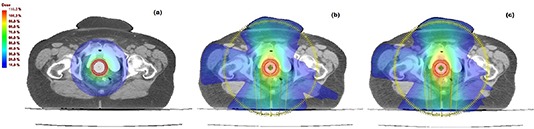
Dose distributions in axial view for the plans developed by (a) HDR‐BRT, (b) AGVMAT1, and (c) AGVMAT2.

**Figure 3:**
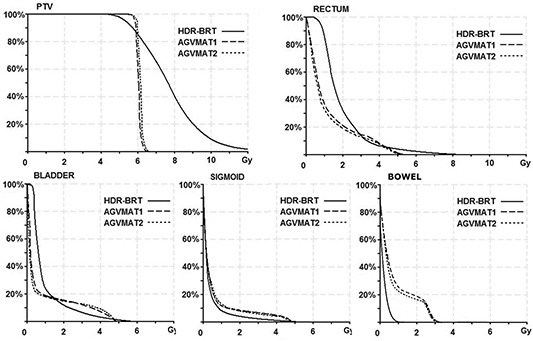
Cumulative DVHs with different techniques for PTV, rectum, bladder, sigmoid, and bowel of a representative case.

Examples of dose distributions are shown in Fig. 2 for axial views. In particular, the coverage of the target volume for plans developed on CT of a representative patient can be seen. Representative DVHs for PTV, rectum, bladder, sigmoid, and bowel are shown in Fig. 3. However, similar dose distribution/DHVs were obtained for the other patients.

The numerical findings in which averages were calculated for the 51 patients and errors indicate interpatient variability at 1 standard deviation level are reported in Table 2.

**Table 2:** Dosimetric and delivery time comparison between AGVMAT and HDR‐BRT.

		*BRT (a)*	*AGVMAT1 (b)*	*AGVMAT2 (c)*	p≤0.01	p≤0.05	p≥0.05
PTV	$d_{\text{mean}}$ (Gy)	1024±252	560±10	568±10	ab, ac		bc
	$d_{\max}$ (Gy)	1721±228	619±22	625±19	ab,ac		bc
	EUD (Gy)	970±234	822±28	827±24	ab,ac		bc
	${\text{mLTCP}}_{1}$	0.54	0	0	ab, ac		bc
	${\text{mLTCP}}_{2}$	0.92	0	0	ab,ac		bc
	${\text{mLTCP}}_{3}$	1	0.63	0.61	ab,ac		bc
	${\text{mLTCP}}_{4}$	1	0.95	0.98	ab,ac		bc
Rectum	$d_{2\text{cc}}$ (Gy)	556±32	498±31	492±35	ab,ac		bc
	$d_{1\text{cc}}$ (Gy)	645±62	528±17	532±19	ab,ac		bc
	$d_{\max}$ (Gy)	852±126	574±17	584±13	ab,ac	bc	
Sigmoid	$d_{2\text{cc}}$ (Gy)	307±144	300±99	298±104			ab,ac,ac
	$d_{1\text{cc}}$ (Gy)	386±95	391±110	397±116			ab,ac,ac
	$d_{\max}$ (Gy)	594±252	487±87	496±78		ab,ac	bc
Bladder	$d_{2\text{cc}}$ (Gy)	504±62	512±34	521±34			ab,ac,ac
	$d_{1\text{cc}}$ (Gy)	578±72	520±31	524±51		ab,ac	bc
	$d_{\max}$ (Gy)	594±124	514±36	519±48	ab,ac		bc
Bowel	$d_{\max}$ (Gy)	165±45	356±92	362±94	ab,ac		bc
Int. dose	$d_{\text{mean}}$ (Gy)	42±13	60±12	65±12	ab,ac	bc	
	T (min)	9.24±3.12	3.76±0.56	4.56±0.82	ab,ac,bc		
	T_BRT_/T	1	2.46	2.03			
	MU/Gy		251±33	304±52			

Statistical significance‐of‐comparison between pairs is indicated by the corresponding pairs of numbers.

BRT=(a); AGVMAT1=(b); AGVMAT2=(c); mLTCP=(minimum) value of local tumour control probability ($r_{0}=0.62\text{mm}$; ${\text{mLTCP}}_{1,2,3,4}=\text{mLTCP}$ ($\rho =10^{7}{\text{cm}}^{3}$, $10^{6}/{\text{cm}}^{3}$, $10^{5}/{\text{cm}}^{3}$, $10^{4}/{\text{cm}}^{3}$); Int. dose=Integral dose; T=average of delivery treatment time; $T_{BRT}/T=$ ratio between HDR‐BRT and average delivery treatment time; MU/Gy= number of monitor units per gray.

**Figure 4:**
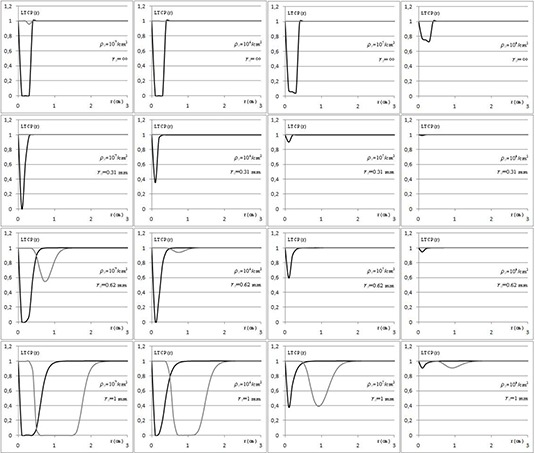
LTCP curves for HDR‐BRT (gray) and AGVMAT (black) with constant ρ (row 1) and variable cell densities (rows 2, 3, and 4) with different values for $r_{0}$ (similar curves were obtained with one or two arcs). Curves were calculated assuming $\rho =10^{7}\text{cells}/{\text{cm}}^{3}$ (column 1) to simulate the clonogens residual in the tumor bed and $\rho_{0}=10^{6}$, $10^{5}$, and $10^{4}\text{cells}/{\text{cm}}^{3}$ (columns 2, 3, and 4, respectively) to simulate different values of subclinical clonogens in the microscopic extension region. The radius r=0.5 cm represents the edge of the CTV. (LTCP values for distances larger than 3 cm are continuously equal to 1.)

LTCP curves corresponding to different values of constant and exponential cell densities by varying $\rho_{0}$ and $r_{0}$ (without errors) are shown in Fig. 4.

### A. target coverage

Averages from DVHs for maximum dose, mean dose and EUD on PTV (which is equal to CTV for HDR‐BRT) are shown in Table 2.

As expected, AGVMAT1 and AGVMAT2 provided a uniform dose distribution, whereas the HDR‐BRT doses were much higher than the prescription through the target (Fig. 1(b)). For this reason, the EUD values of HDR‐BRT were always higher than the AGVMAT.

Also, the minimum values of LTCP obtained with HDR‐BRT were significantly higher than those obtained with AGVMAT curves, being comparable only in the case of low steepness cell density (higher probability of tumor control). This means that, in all the clinical range of clonogen density analyzed, the therapeutic results of HDR‐BRT could not be reproduced by the uniform dose distribution from AGVMAT without increasing the dose prescribed. In other words, this approach predicts a lower TCP employing AGVMAT rather than HDR‐BRT, assuming the same prescribed dose. An obvious explanation could be the high gradient generated using brachytherapy sources. However, a similar distribution should be obtained using AGVMAT, but with some limitations due to the increase of arc modulation and of MUs.

### B. organs at risk

Table 2 shows the results from DVH analysis with important differences between plan methods for the OARs. In particular for the rectum, the AGVMAT doses of $d_{2\text{cc}}$, $d_{1\text{cc}}$ and $d_{\max}$ were significantly reduced (p<0.01) with respect to HDR‐BRT. These results are expected to be clinically significant when compatible with the identical therapeutic results of CTV.^(^ ^24^ ^)^

The sigmoid AGVMAT averages of $d_{2\text{cc}}$ and $d_{1\text{cc}}$ were comparable with those obtained by HDR‐BRT, while no significant differences were found for maximum doses. However, these values were far from the dose tolerance.

The bladder averages of $d_{2\text{cc}}$ in HDR‐BRT were lower than those obtained by AGVMAT, with a difference significant as trend. This trend was reversed in the case of $d_{1\text{cc}}$ and maximum doses, with a moderate statistical significance (p<0.05).

The averages of $d_{\max}$ for the bowel were significantly lower in HDR‐BRT than AGVMAT.

The integral dose accumulated in the healthy tissue area surrounding the region of the vaginal vault was evaluated by extracting the average dose of the DVH considered as a surrogate of the integral dose itself. Although these mean doses were very low in both cases, due to the small volume of the vaginal vault, the AGVMAT mean doses were proportionally higher than those of HDR‐BRT with considerable significance.

### C. Monitor units and delivery time

The averages of MU/Gy were 251±33 for AGVMAT1 with an average dose rate of DR=400 MU/min, corresponding to an expected average beam‐on time of 3.76±0.56 min per fraction of 6 Gy. In AGVMAT2, because of two arcs, the number of MU per Gy was higher than AGVMAT1—MU/Gy=304±52, corresponding to an expected average beam with a time of 4.56±0.82 min for each fractions of 6 Gy. Thus, the ratio of average BRT delivery time (TBRT) and AGVMAT beam‐on time (T) was about two or three times higher between AGVMAT1 and AGVMAT2 (Table 2).

## IV. DISCUSSION

Usually, the radiation therapy of the vaginal vault in patients affected by GC after hysterectomy consists of a combination of prophylactic external beam radiation and a brachytherapy boost with prescription at a distance of 0.5 cm from the vaginal cylinder surface. External beam and brachytherapy components of treatment are planned independently of each other. However, these treatment modalities have somewhat complementary strengths and weaknesses.

External beam (i.e., by VMAT) produces a more homogeneous dose distribution, while brachytherapy avoids much of the geometric uncertainty characteristic of current external beam delivery techniques, such as intensity‐modulated techniques. “Combining these two modalities provides new opportunities for improving well‐established brachytherapy/external beam regimens, but also expands the possibilities for delivering more aggressive radiotherapy regimens to extended locoregional target volumes.”^(^ ^25^ ^)^

Therefore, according to Williamson,^(^ ^25^ ^)^ in this work we evaluated a combination of EBRT and BRT techniques — the applicator guided volumetric arc therapy technique — as an alternative to high‐dose‐rate brachytherapy, employing radiobiological and dosimetric tools of comparison.

Our results showed that the rectal strip included in the PTV was subjected to an increasing dose profile starting from the dose prescribed when planned by HDR‐BRT, while, due to a more uniform PTV coverage, the same strip was subjected to a dose rather similar to the prescription dose, when planned by AGVMAT.

For this reason, the AGVMAT plans were able to keep the maximum dose on rectum lower than the HDR‐BRT. This happened also because the vaginal cylinder used in the AGVMAT repositioning phase, had a double role: first, as an immobilization device which permitted a better reproducibility of plans; second, because being clearly visible in cone‐beam CT, it reduced the interfraction setup errors.

On the other hand, while doses from AGVMAT or HDR‐BRT were very similar for the bladder as well as for the sigmoid, of relevance, the HDR‐BRT was able to reduce the doses to the bowel (brachytherapy is currently adopted as boost for bowel sparing).

Also, the mean integral doses were comparable between techniques. This might be due to the small size of the vaginal vault notwithstanding the notable differences between techniques in terms of dose distribution — more similar to a dish with AGVMAT (because the coplanarity of arcs), more spherical with HDR‐BRT (because the radial distribution from the center of ${\;}^{192}\text{Ir}$ sources to the outside).^(^ ^26^ ^)^ Hence, while low doses in larger volumes were observed using AGVMAT, high doses in small volumes and very low doses in larger volumes were obtained by HDR‐BRT. However, whether “a lot to a little” or “a little to a lot” is better in terms of integral dose^(^ ^27^ ^,^ ^7^ ^)^ remains a controversial issue as highlighted by Zaider et al.^(^ ^27^ ^)^ and confirmed by Aydogan et al.,^(^ ^7^ ^)^ and requires further investigation. Moreover, as was expected, a reduction in terms of beam‐on time was obtained with AGVMAT with respect to HDR‐BRT. This is certainly a positive point for the patients but could be critical, in terms of tumor control, when clonogenic cells with very short repopulation times are considered.

It remains to be established whether the uniform dose on target obtained by AGVMAT would give the same therapeutic results as HDR‐BRT. An initial evaluation was done by using the EUD that resulted significantly higher in HDR‐BRT with respect to AGVMAT. However, the EUD represents an average of values generated by cells exposed to different doses, although on a biological basis. Therefore, to better investigate the actual equivalence in terms of therapeutic efficacy of techniques, a new tool of analysis has been introduced: the LTCP. The latter enables the evaluation of the local value of TCP taking into consideration the spatial variability of the clonogenic cell density, which is assumed to be constant in the target while decreasing exponentially in the microscopic extension region.

Webb and Nahum^(^ ^18^ ^)^ have stated that if the clonogenic cell density is constant throughout the tumor, a uniform dose distribution will produce the highest TCP for a fixed energy deposition. Our results showed a significantly higher LTCP in HDR‐BRT than AGVMAT, while comparable values were found only in the case of low steepness cell density. In particular, with respect to the different values of constant clonogen density analyzed, lower values of LTCP were obtained based on the uniform dose distribution generated using AGVMAT rather than HDR‐BRT. As the TCP increases with the prescribed dose, the same TCP (which is a surrogate of expected therapeutic results in terms of tumor control) of HDR‐BRT could be obtained in AGVMAT, increasing the dose prescribed. Unfortunately, this could nullify the advantages obtained in terms of sparing for the rectum.

## V. CONCLUSIONS

In this study, a dosimetric and radiobiological comparison between the applicator‐guided volumetric‐arc therapy and the high‐dose‐rate brachytherapy treatment of the vaginal vault was made for patients with GC. When the high reproducibility of positioning (due to the vaginal applicator commonly used in brachytherapy) and an accurate internal organ registration were assumed, the applicator‐guided volumetric‐arc therapy seemed to have the potential for improving critical structure avoidance, as demonstrated by a reduced rectal dose. However, brachytherapy still performed better in terms of PTV coverage because of the greater EUD and better LTCP curves.
